# Uso de los métodos de diagnóstico de relación esquelética en los artículos publicados del 2018 al 2020 en las cuatro revistas más importantes de ortodoncia

**DOI:** 10.21142/2523-2754-0901-2021-041

**Published:** 2021-03-11

**Authors:** Enrique Manuel De los Ríos Fernández

**Affiliations:** 1 Facultad de Odontología, Universidad Nacional de Tucumán. Tucumán, Argentina. enrique78@hotmail.com Universidad Nacional de Tucumán Facultad de Odontología Universidad Nacional de Tucumán Tucumán Argentina enrique78@hotmail.com

**Keywords:** diagnóstico, relación esquelética, Diagnosis, Skeletal relationship

## Abstract

**Objetivo:**

Determinar el método de diagnóstico de relación esquelética más empleado, según las cuatro revistas más importantes de ortodoncia en el periodo 2018-2020.

**Metodología:**

Se elaboró un análisis documental de información compilada ya existente referida a los procedimientos de diagnóstico utilizados con mayor frecuencia en el reconocimiento de las relaciones esqueléticas en las cuatro revistas más importantes de ortodoncia desde el año 2018 hasta el 2020, según SCImago 2018, cuyo soporte en internet proporciona una serie de parámetros acerca del impacto de las publicaciones y revistas. Con esa finalidad, se realizó una sinopsis de la información analizada para establecer una correlación de las fuentes y hacer cotejos entre ellas de manera crítica. La exploración bibliográfica virtual de bases de datos se realizó esencialmente a través de Medline; se evaluó y seleccionó la bibliografía, organizada según la trascendencia y la índole científica; y, luego, se procesaron los resultados para responder las demandas de la investigación.

**Resultados:**

En la revista AJO-DO, el 92,2% utiliza el método de ANB y el 7,8% no describe el método utilizado. En la revista AO, el 100% refiere el uso del ANB. Finalmente, en las revistas EJO y KJO, el 93,8 % y el 95% utilizaron el ANB, respectivamente.

**Conclusión:**

El método más frecuentemente utilizado para la determinación de la relación esquelética es el ANB, aun cuando este indicador presenta un riesgo de efecto geométrico, ya que es un método ampliamente conocido.

## Abstract (en)

**Objective:**

To determine the skeletal-related diagnostic methods most commonly used according to the four most important orthodontics journals in the period from 2018 to 2020.

**Methods:**

A review of the data available regarding the diagnostic procedures most frequently used to determine skeletal relationships in the four most important orthodontics journals from 2018 to 2020. Article review was performed using SCIMAGO 2018, which provides a series of parameters ranking the importance of the impact of publications and journals. We made a synopsis of the information analyzed to establish a correlation and compare sources, and critically examined the articles collected on the subject of interest. An online bibliographic search of databases including mainly Medline was carried out, followed by evaluation and selection of the bibliography, organized according to the significance and nature of the study.

**Results:**

The four journals included in the bibliographic search were: AJODO, AO, EJO and KJO. The A point-Nasion-B point (ANB) method was used in 92.2% of the articles published in AJODO, and 7.8% did not describe the method used. Twenty-three articles in the AO journal were evaluated, all of which referred to the use of ANB as the method used. Finally, 16 and 11 articles from the EJO and KJO journals, respectively, were evaluated, with 93.8% and 95%, respectively of the articles using ANB as the method of choice for determining skeletal relationships.

**Conclusion:**

The method of choice for skeletal-related diagnostics is ANB, although this widely known method presents a risk of geometric effect.

## INTRODUCCIÓN

El propósito central de los procedimientos en ortodoncia es mejorar la estructura esquelética, dental y de los tejidos blandos del paciente ^1^^,^^2^. La clase esqueletal define la relación en sentido anteroposterior de los maxilares ^3^, además de su relación con las demás estructuras óseas y tejidos blandos ^4^, por lo que coexisten factores dentarios y óseos que determinan las relaciones esqueletales ^5^ y exteriorizan particularidades constitutivas inherentes de cada clase esqueletal, las cuales son el producto de manifestaciones genéticas expresadas a través del crecimiento y el desarrollo ^6^. Estas particularidades estructurales inherentes de cada tipo esqueletal esclarecen la presencia de adecuaciones funcionales en relación con la bioestructura, la deglución, la masticación, la respiración y el habla ^7^. 

El sistema estomatognático en personas con clase I esqueletal tiene como característica bases óseas equilibradas y funcionalmente normales ^8^, mientras que en sujetos con clases II y III se observa una falta de equilibrio estructural que puede predisponer a que se presenten modificaciones funcionales, como es el caso de la fonoarticulación ^(9, 10)^.

En ortodoncia, el diagnóstico es un procedimiento que incluye la sinopsis de los datos obtenidos luego de una evaluación clínica exhaustiva ^11^, para obtener una evaluación presuntiva, la cual permite trazar un correcto plan de tratamiento ^(12, 13)^, pero para ello se debe tener en cuenta una serie de circunstancias como edad, sexo, raza, biotipo facial, discrepancias dentomaxilofaciales y predicciones de desarrollo ^14^^-^^16^.

En la actualidad, existen muchas publicaciones y gran parte de las investigaciones elaboradas por maestros e investigadores son difundidas a través de artículos de revistas o de libros ^17^. En estos textos se aplican diversos métodos de diagnóstico para la identificación de las relaciones esqueléticas ^18^. La sistematización de los métodos empleados durante los últimos veinte años permitirá conocer cuáles fueron los más utilizados como resultado de la actividad científica e investigativa ^19^^-^^21^.

En cuanto a su relevancia respecto del área clínica, esta se relaciona con la información que se proporcionará, teniendo en cuenta el año de la publicación y el lugar en el que se realizó, lo cual aportará una perspectiva más amplia al momento de emitir un correcto diagnóstico ^22^. La relevancia contemporánea se manifiesta en la importancia de este estudio para llegar a un adecuado diagnóstico, prevención y tratamiento de las diferentes relaciones esqueléticas, según las cuatro revistas más importantes sobre ortodoncia ^23^^,^^24^.

## MATERIALES Y MÉTODOS

Se elaboró un análisis documental, de información compilada ya existente, referida a los procedimientos de diagnóstico utilizados con mayor frecuencia para el reconocimiento de las relaciones esqueléticas en las cuatro revistas más importantes de ortodoncia desde el año 2018 hasta el 2020, según SCImago 2018, cuyo soporte en internet proporciona una serie de parámetros acerca del impacto de las publicaciones y revistas.

Se incluyeron en el estudio artículos originales que involucran el diagnóstico de relaciones esqueléticas cefalométricamente, publicaciones comprendidas entre los años 2018 al 2020, de las cuatro revistas más importantes de ortodoncia. Se excluyeron revisiones sistemáticas, cartas al editor, editoriales, reportes y series de caso.

Posteriormente, se llevó a cabo una sinopsis de la información analizada para establecer una correlación de las fuentes y hacer cotejos entre ellas de manera crítica. Para ello se efectuó la exploración bibliográfica virtual de bases de datos, esencialmente a través de Medline; se evaluó y seleccionó la bibliografía; se organizó según la trascendencia y la índole científica; y, luego, se procesó los resultados para responder las demandas de la investigación, considerando el problema a estudiar, las concepciones esenciales, las conjeturas y modelos que aplican los autores, las deducciones y productos de sus investigaciones, los cotejos de sus conclusiones con el resto de publicaciones y el aporte de esta información a la presente investigación. Finalmente, se realizó un análisis de los resultados y las conclusiones para dar respuesta a la pregunta de investigación.

### Plan de análisis

Se analizaron los resultados con ayuda del programa estadístico SPSS versión 25, empleando la prueba de chi cuadrado para valorar asociaciones entre el tipo de revista y la aplicación de los métodos de medición de la relación esquelética, así como las características de las publicaciones. Se trabajo a un nivel de significancia de p < 0,05.

## RESULTADOS

En referencia a los métodos elegidos para la determinación de la relación esquelética, de acuerdo con las revistas de ortodoncia analizadas, la Tabla 1 muestra que, de los 51 artículos publicados en la revista American Journal of Orthodontics and Dentofacial Orthopedics (AJO-DO), el 92,2 %, utiliza el método de ANB y el 7,8% no describe el método utilizado. En el caso de la revista American Orthodontics (AO), con 23 artículos evaluados, el 100% refiere el uso del ANB. Las revistas European Journal of Orthodontics (EJO) y Korean Journal of Orthodontics (KJO), con 16 y 11 artículos evaluados, mostraron resultados del 93,8 % y el 95% en el uso del ANB, respectivamente. No se encontró asociación significativa entre el método utilizado y la revista evaluada p = 0,197.

**Tabla 1:** Método elegido para la determinación de la relación esquelética en los artículos evaluados en cada una de las revistas de ortodoncia analizadas

Revista	Método					
		No describe	ANB	Wits	Total
AO	n	0	23	0	23
	%	0,00%	100,00%	0,00%	100,00%
AJO-DO	n	4	47	0	51
	%	7,80%	92,20%	0,00%	100,00%
EJO	n	0	15	1	16
	%	0,00%	93,80%	6,30%	100,00%
KJO	n	1	10	0	11
	%	9,10%	90,90%	0,00%	100,00%
Total	n	5	95	1	101
	%	5,00%	94,10%	1,00%	100,00%

p = 0,197

Prueba de chi cuadrado

De los 101 artículos evaluados, la Tabla 2 muestra que 95 de ellos usó como primer método para la determinación de la relación esquelética el ANB, lo que corresponde a un 94,1%, 5 no especificaron el método empleado (5%) y solo uno utilizó el análisis de Wits (1%). Asimismo, se evidencia que, entre los métodos complementarios especificados en los artículos, el de segunda elección fue Wits, empleado en 24 de ellos, lo que corresponde a un 23,8%.

**Tabla 2:** Métodos elegidos en un mismo artículo para la determinación de la relación esquelética en los artículos evaluados

Primer método de elección para evaluar la relación esquelética		
Método	n	%
No describe	5	5,0
ANB	95	94,1
Wits	1	1,0
Total	101	100,0
Segundo método complementario al primero de elección para evaluar la relación esquelética		
No describe	77	76,2
Wits	24	23,8
Total	101	100,0

Respecto de los métodos elegidos para la determinación de la relación esquelética de acuerdo con el año de publicación (2018, 2019 y 2020), la Tabla 3 muestra que, independientemente del año de publicación, el método más elegido fue el ANB, seguido en menor porcentaje por el método de Wits (p = 0,173).

**Tabla 3:** Métodos elegidos para la determinación de la relación esquelética de acuerdo con el año de publicación de los artículos evaluados

Revista	Método					
		No describe	ANB	Wits	Total
2020	n	2	21	1	24
	%	8,30%	87,50%	4,20%	100,00%
2019	n	3	37	0	40
	%	7,50%	92,50%	0,00%	100,00%
2018	n	0	37	0	37
	%	0,00%	100,00%	0,00%	100,00%
Total	n	5	95	1	101
	%	5,00%	94,10%	1,00%	100,00%

p = 0,173

Prueba de chi cuadrado

A partir de los métodos elegidos para la determinación de la relación esquelética según la procedencia del artículo, la Tabla 4 muestra que, mayoritariamente, el método elegido es el ANB, aunque se observa que unos pocos artículos procedentes de Sudamérica y Asia no describen el método y solo un artículo, procedente de Sudamérica, utiliza el análisis de Wits (p = 0,034).

**Tabla 4:** Métodos elegidos para la determinación de la relación esquelética según la procedencia del artículo evaluado

Revista	Método					
		No describe	ANB	Wits	Total
Norteamérica	n	0	16	0	16
	%	0,00%	100,00%	0,00%	100,00%
Sudamérica	n	2	8	1	11
	%	18,20%	72,70%	9,10%	100,00%
Europa	n	1	21	0	22
	%	4,50%	95,50%	0,00%	100,00%
Asia	n	2	50	0	52
	%	3,80%	96,20%	0,00%	100,00%
Total	n	5	95	1	101
	%	5,00%	94,10%	1,00%	100,00%

p = 0,034

Prueba de chi cuadrado

## DISCUSIÓN

Los métodos empleados en las mediciones cefalométricas que se mencionan presentan numerosas dificultades para su interpretación, fundamentalmente cuando queremos vincular anteroposteriormente el maxilar con la mandíbula. La información hallada en la presente investigación toma en cuenta el cuestionamiento de muchos profesionales debido a las controversias sobre la utilización del ANB y el Wits, con el argumento de su escasa confiabilidad y limitado soporte científico en los estudios cefalométricos con fines ortodóncicos, en parte la variación del ángulo ANB. Esto se puede atribuir a otros factores aparte de las diferencias sagitales de la base apical, como la rotación del plano silla-nasión, la rotación de los maxilares y el largo del plano silla-nasión. ^26^^-^^29^. 

Otros autores ^30^^-^^32^ indican que las mediciones angulares que se realizan tomando como referencia la línea S-N no son del todo confiables. El punto nasion está ubicado en el límite externo de la sutura frontonasal y no en la propia base, lo que lo hace susceptible de sufrir cambios de remodelación con el crecimiento, considerando que, si bien la migración de este punto dentro del plano silla-nasión va hacia adelante, siguiendo la dirección del mismo, en algunos casos puede ir en dirección vertical, lo que origina una incorrecta impresión de la forma en que crece la cara. 

Es de suma importancia reconocer las variaciones que sufren los ángulos involucrados al medir las relaciones maxilares con relación al plano silla-nasión, por su posición, por la ubicación del punto nasión en particular y por la impresión incorrecta que nos enmascara las relaciones verdaderas. 

Se planteó el presente trabajo, en el que se analizaron todos los artículos utilizados en la identificación de las relaciones esqueléticas en las cuatro revistas más importantes de ortodoncia publicadas del 2018 al 2020. Llama la atención que en la mayoría de los artículos analizados (92,2%) se utilice el ANB teniendo en cuenta su poco sustento científico y confiabilidad en estudios cefalométricos, independientemente del año (p = 0,173) y lugar de publicación de la revista (p = 0,034). Estos datos indican que el uso del ANB es el más extendido, ya que evalúa de manera sencilla la diferencia entre los dos ángulos anteriores y establece la relación entre maxilar y mandíbula en sentido anteroposterior, por lo que es uno de los indicadores para el diagnóstico diferencial de clase esquelética/dentaria. 

Por su parte, el análisis de Witz, según Jacobson, define el grado de desarmonía esquelética entre las bases apicales con un índice de confianza superior al ángulo ANB, ya que los puntos de referencia empleados (A y B) se encuentran localizados en las propias bases apicales y no en la distancia. Pese a esto, no es un método muy usado, según lo hallado en el presente estudio. 

También se realizaron estudios para medir la discrepancia esqueletal con el ángulo beta ^33^, en los que se le comparó con los valores para caucásicos y se correlacionaron con ANB y Wits. Se concluyó que el ángulo beta es altamente confiable para evaluar las discrepancias sagitales de maxilar y mandíbula, ya que la media y desviación estándar para cada clase esqueletal son estadísticamente diferentes entre ellas. Por tanto, es igual o más fiable que el ANB y Wits por excluir a estructuras craneales o planos oclusales en su elaboración, y basarse en puntos localizados únicamente en los maxilares; sin embargo, no se encontró ninguna publicación que haya empleado este método.

## CONCLUSIONES

El método más frecuentemente utilizado para la determinación de la relación esquelética es el ANB, aun cuando presenta un riesgo de efecto geométrico, ya que es un método ampliamente conocido y de interpretación. Los otros métodos, a pesar de tener aparentemente una mayor precisión, no se hallan tan difundidos, lo cual hace que su uso para el diagnóstico de las discrepancias sagitales sea menor.

## Fuente de financiamiento:

Autofinanciado
